# On-chip label-free cell classification based directly on off-axis holograms and spatial-frequency-invariant deep learning

**DOI:** 10.1038/s41598-023-38160-3

**Published:** 2023-07-31

**Authors:** Matan Dudaie, Itay Barnea, Noga Nissim, Natan T. Shaked

**Affiliations:** Department of Biomedical Engineering, Faculty of Engineering, Tel Aviv University, 69978 Tel Aviv, Israel

**Keywords:** Biomedical engineering, Cancer imaging

## Abstract

We present a rapid label-free imaging flow cytometry and cell classification approach based directly on raw digital holograms. Off-axis holography enables real-time acquisition of cells during rapid flow. However, classification of the cells typically requires reconstruction of their quantitative phase profiles, which is time-consuming. Here, we present a new approach for label-free classification of individual cells based directly on the raw off-axis holographic images, each of which contains the complete complex wavefront (amplitude and quantitative phase profiles) of the cell. To obtain this, we built a convolutional neural network, which is invariant to the spatial frequencies and directions of the interference fringes of the off-axis holograms. We demonstrate the effectiveness of this approach using four types of cancer cells. This approach has the potential to significantly improve both speed and robustness of imaging flow cytometry, enabling real-time label-free classification of individual cells.

## Introduction

On-chip image-based classification of cells is an essential tool in cell analysis for pathology, profiling, and diagnosis of various types of cells. Imaging flow cytometry is typically performed with cell labeling^1–4^, with the risk of damaging the cell viability. In contrast, label-free imaging flow cytometry^5,6^ presents a more natural approach and is based on using an internal contrast mechanism of the cells rather than external chemical labeling. Machine-learning algorithms can be used for cell classification. This is typically done by extracting hand-crafted features and then plugging them into a statistics-based discrimination algorithm, such as gradient boosting, support vector machine (SVM), or a fully connected neural network, as a supervised learning framework for classification and identification of the cells^7–13^. Alternatively, convolutional neural networks (CNNs) can be used as the supervised learning framework while running it on the cell images directly, without feature extraction, with the advantage of taking into account hidden features that might be missed when extracting hand-crafted features from the images^14–18^. Collecting the image database for training and testing the classifying network in real-time is not an easy task, as there is a need to balance the throughput, flow rates, imaging rate, and computational time^19,20^.

Digital holography can retrieve the quantitative cell phase profile taking into consideration both its morphology and content^21,22^, and combined with machine learning, can be used for cell classification^23–26^. In digital holography, a clean copy of the illuminating beam (i.e., a reference beam) interferes with the beam that has passed through the sample, creating a hologram on the digital camera. The quantitative phase profile of the cell can then be calculated per each image pixel, which is proportional to the optical phase delay (OPD) of the cell. The OPD is equal to the product of the cell thickness and the difference between the integral refractive indices of the cell and the surrounding medium. In off-axis holography, by introducing a small angle between the sample beam and the reference beam, the OPD can be reconstructed from a single holographic image. Thus, making high-speed holographic imaging of flowing cells possible. However, the calculation time of the OPD is a limiting factor, so the analysis is typically done by recording the off-axis holograms first and performing the analysis offline.

In contrast to classification based on the OPD profile of the cell, classification based on the holograms directly enables a faster response time of the system since no time-consuming computations are needed to calculate the OPD and features prior to the classification. In addition, classification based on the holograms enables taking into account the amplitude profile of the cell as well. In off-axis holograms, the most dominant factor is the interference fringes. Priscoli et al.^27^ have previously shown classification between two neuroblastoma cell lines based on the raw off-axis holograms. However, they did not take into consideration fringe frequency variations. The angle between the sample and reference beams determines the frequency and orientation of the fringes. These are highly sensitive to small changes in the system environment and may vary between experiments and even during an experiment.

In this paper, we propose a spatial-frequency-invariant deep neural network that can perform cell classification directly on the off-axis holograms, which is an important step toward real-time label-free cell cytometry based on off-axis digital holographic microscopy. We demonstrate the new direct classification ability on off-axis holograms with variable spatial frequency, created in a semi-synthetic procedure, for four pairs of isogenic cancer cells, and then used the trained network, after transfer learning, to classify experimental holograms of two types of cancer cells during flow in a microfluidic channel chip.

## Materials and methods

### Cell types and sample preparation

We imaged and analyzed four isogenic cell lines, termed SW^28,29^, HS, TE, and WM. The SW cells are a pair of isogenic cancer cell lines: SW480 (ATCC CCL-228)—colorectal adenocarcinoma cells, and SW620 (ATCC CCL-227)—a metastatic form of these cancer cells collected from a lymph node of the same patient. The HS cells are a pair of a normal skin cell line, HS895.Sk (ATCC CRL-7636) and a melanoma cancer cell line, HS895.T (ATCC CRL-7637), taken from the same patient. The TE cells are a pair of a normal skin cell line, TE353.Sk (ATCC CRL-7761), and a melanoma cancer cell line, TE354.T (ATCC CRL-7762), are both taken from the same patient. The WM cells are a pair of a melanoma skin cell line, WM115 (ATCC CRL-1675), and a metastatic melanoma skin cell line, WM266.4 (ATCC CRL-1676), both taken from the same patient.

All the cells were grown in Dulbecco's modified Eagle medium (ATCC, SN. 30-2002), supplemented with 10% fetal bovine serum (BI, SN. 04-007-1A). 2 mM L-glutamine (BI, SN. 03-020-1B) were added for the WM cell lines. The cells were incubated under standard humidity and temperature of 37 °C with 5% CO_2_ until 80% confluence was reached. Before imaging, the cells were trypsinized for suspension and supplemented with a suitable medium.

### Off-axis holographic imaging flow cytometry system

**Figure 1:**
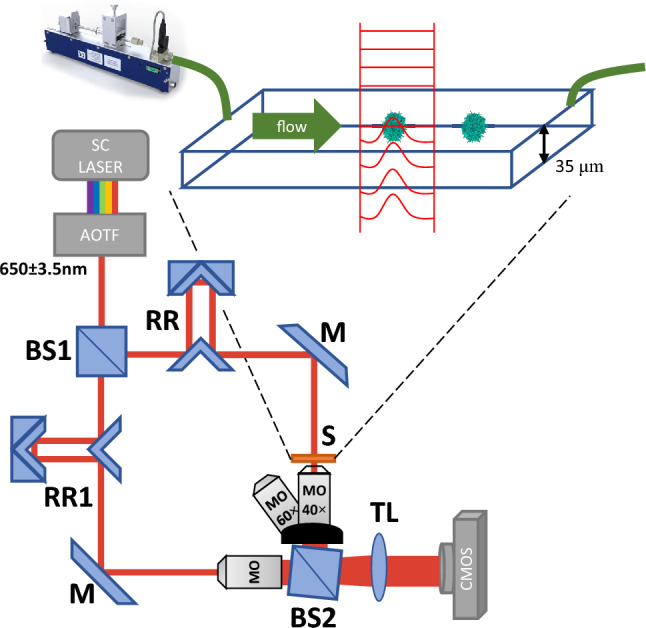
Label-free imaging flow cytometry setup used in the experiment. The setup is based on a Mach–Zehnder interferometer and a microfluidic channel, shown in the sketch above. The laser wavefront is illustrated in red. SC, supercontinuum laser source; BS1 and BS2, beam splitters; RR and RR1, retroreflectors; S, sample; MO, microscope objective; M, mirror; TL, tube lens. CMOS, digital camera.

The cells' off-axis holograms were acquired using a custom-built Mach–Zehnder interferometer microscope, as depicted in Fig. 1. The Illumination laser was a low-coherence supercontinuum laser (Fianium, SC-400-4) coupled to an acousto-optical tunable filter (AOTF) set and emits a 650 ± 3.5 nm beam. The beam is split into two by a beam-splitter (BS1). One of the beams is directed with a mirror (M) to pass through the sample chip (S), modulating the imaged cell phase onto the light wavefront and through a microscope objective (MO, Newport 60×, 0.85 NA or Leica 440 40×, 0.66 NA). The other beam passes through a duplicate objective. The optical path of the beams is aligned to match by moving a retroreflector (RR1) accordingly. Both beams are combined with a second beam-splitter (BS2) through a tube-lens (TL, with a focal length of 150 mm or 200 mm with respect to the objectives) and interfere at the image plane at a small off-axis angle, where a CMOS camera (Thorlabs CC1545M), with 5.2 μm pixel size, records the image.

Still images were captured by using an adhesive chamber (Scureseal, Grace Bio-labs, 800 μm depth) on a coverslip and refocusing the sample by moving the coverslip along the optical axis for each cell. While imaging a dynamic video, the cell can flow in and out of focus. To manage this problem, we used a shallow microchannel with a depth and width of 35 μm × 700 μm^30,31^, respectively. The shallow height reduces out-of-focus occurrences. The flow was generated by a pump (CETONI Syringe Pump neMESYS 290N) with low flow rates of 7–30 μl/hr. This rate allows the camera to utilize the entire field-of-view at a framerate of approximately 20 frames per second.

### Dataset preparation

From the recorded holograms, we cropped out 290 × 290-pixel single-cell images, as dictated by the largest cell in the dataset. The experimental cell dataset size was 923 and 704 for the SW480 and SW620 cells, respectively; 127 and 138 for the HS895.Sk and HS895.T cells, respectively; 169 and 170 for the TE353.Sk and TE354.T cells, respectively; and 105 and 183 for the WM155 and WM266.4 cells, respectively. Each hologram went through an OPD reconstruction algorithm to reconstruct the OPD and the amplitude profiles recorded within the hologram, as follows: the 2-D Fourier transform of the hologram is calculated, one of the cross-correlation terms is cropped and undergoes an inverse 2-D Fourier transform. The resulting complex matrix is divided by a background complex wavefront matrix, produced by the same procedure, from a background hologram without any cells. Resulting in the complex wavefront (CWF) of the sample defined as:

1 $$CWF\left( {x,y} \right) = Amplitude\left( {x,y} \right) \cdot \exp \left( {i\frac{2\pi }{\lambda }OPD\left( {x,y} \right)} \right),$$

where $\lambda$ is the central laser wavelength. For creating the training dataset, using the experimentally acquired CWF of each cell, we synthetically prepared new digital off-axis holograms with various off-axis spatial frequencies. This was done synthetically by adding the CWF of the cells with a reference wavefront and taking the absolute value square while setting the off-axis angle $\alpha$ and the fringes direction angle $\varphi$, defining the final off-axis synthetic hologram as:

2 $$\left| {e^{{i\frac{2\pi }{\lambda } \cdot \sin \left( \alpha \right) \cdot \left( {x\cos \left( \varphi \right) + y\sin \left( \varphi \right)} \right)}} + CWF\left( {x,y} \right)} \right|^{2} .$$

The off-axis angle $\alpha$ was chosen within a limit to resemble an authentic off-axis hologram and is related to the fringe frequency as $f=\frac{\mathrm{sin}\alpha }{\lambda }$. The lower boundary of the frequency is set so that the cross-correlation terms of the hologram would not overlap the zero term in the Fourier plane, i.e., the lowest frequency of the point spread function (PSF) would not be less than $3/2$ of the PSF size. The minimum spatial frequency is then:

3 $$f_{\min } = \frac{2}{3PSF} = \frac{2NA}{{3\lambda M}},$$

where ${M}$ is the magnification, and $NA$ is the numerical aperture of the objective lens. The upper-frequency boundary is dictated by the Nyquist frequency, calculated to be at least three pixels per cycle of the fringes. For example, with 40 × magnification and NA 0.66, the angle boundaries are $\alpha =[0.63^\circ ,2.38^\circ ]$. The angle parameter $\varphi$ boundaries are between 0 and 180° to avoid repetition of the fringe direction.

We increased the size of the image dataset by 4 and 8 times using image augmentations (flip up-down, flip left–right, rotation in 90°, 180°, 270°, and combinations of flipping and rotation in 90°).

**Figure 2:**
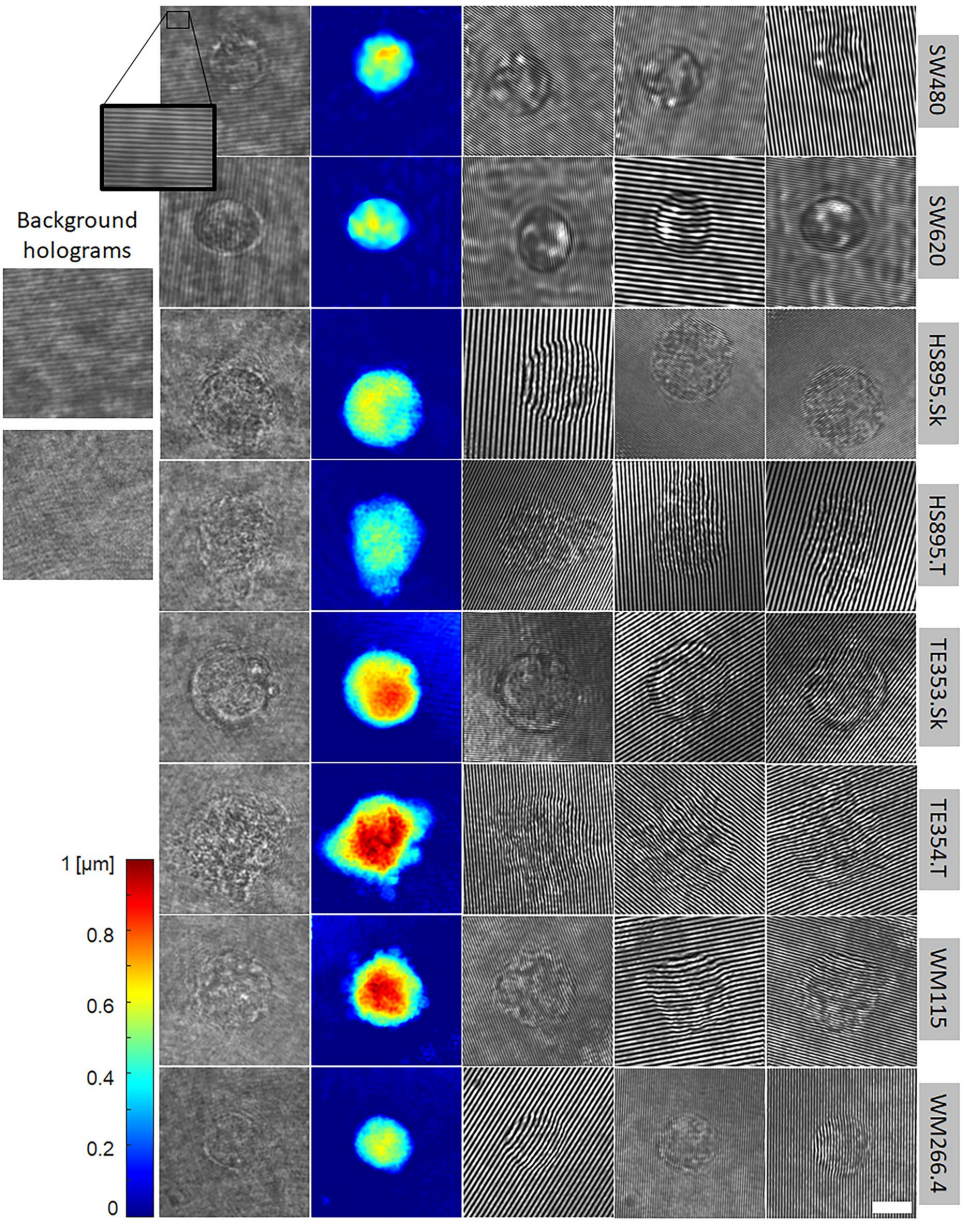
Cell image examples for the various cell types acquired. Left column: experimentally captured off-axis holograms, with background holograms shown on its left. Second column: OPD profiles retrieved from the off-axis hologram. Three right columns: semi-synthetic off-axis holograms generated from the coinciding OPD profiles with different augmentations and random $\alpha$ and $\varphi$ angles, resulting in fringes with various spatial frequencies and directions. These holograms were used for training the deep neural network for cell classification. The white scale bar indicates a length of 10 μm. The color bar on the bottom left refers to the OPD profiles (second column from left) and represents OPD values.

The new off-axis holograms were each normalized to be in the range of [0,1] and saved as a 24-bit PNG image file. The OPD profiles were normalized to the maximum value of the whole dataset and colorized using colormap "Jet". Examples from the dataset are shown in Fig. 2.

### Deep neural network training and testing

After augmentations, the datasets contained 6,508 images for the SW pair, 2,120 images for the HS pair, 2,712 images for the TE pair, and 2,304 images for the WM pair. Each dataset was divided randomly into 80% for training, 10% for validation, and 10% for testing.

Several common CNNs were examined on our dataset (Google net, Resnet18, Resnet50, VGG16, VGG19, and MobileNetV2), where MobileNetV2^32^ outperformed the rest. We used a pre-trained network based on the ImageNet dataset, included in MATLAB 2022b Deep Network Designer toolbox, and changed the input and final layers to fit two-class classification. We used Adam optimizer, with settings of 0.005 constant learning rate and batch size of 64.

On average, the networks started to converge within 3.5 epochs, and the maximum number of epochs was set to 20. The output network was chosen to give the best validation-to-loss value. All the training and testing were done on a parallel CPU (Intel i7 10700).

We trained 30 different MobileNetV2 networks, elaborated below as Experiments 1–30, with different settings, as elaborated in Table 1. To test the network ability to perform under different off-axis fringe spatial frequencies, we carried out eight tests only on the SW pair, with different off-axis angles ($\alpha$) across its boundaries with jumps of 0.25° and constant fringe direction angle $\varphi =0^\circ$. Next, we trained on images with constant off-axis angle $\alpha$ and random $\varphi$ per each image. After proving the network ability to classify directly on off-axis holograms with a constant angle, we tested its ability to classify images with random $\alpha$ and constant $\varphi =0^\circ$, and on both random $\alpha$ and $\varphi$. For the latter case, we increased the size of the dataset by using all eight augmentations on the SW cells. Following those experiments, we used the pre-trained SW network as the basis for training on the other couples of cells with both random off-axis fringe spatial frequencies and angles. For comparison, we trained and tested the CNN on the OPD profiles of the cell pairs. Finally, we verified on the experimental SW-cell holograms the CNN ability for classification on real-world data.

**Table 1:** List of the experiments done on the synthetic holograms and the resulting performance metrics.

Experiment number	Cell pair	Final validation accuracy (%)	Test accuracy (%)	Test recall (%)	Test specificity (%)	Test precision (%)	AUC
1–8^a^	SW	96.86 ± 0.68	96.41 ± 0.86	96.39 ± 1.50	96.55 ± 1.86	97.36 ± 1.49	0.993 ± 0.34
9–16^b^	SW	94.79 ± 0.89	94.67 ± 0.80	93.68 ± 1.36	96.18 ± 1.93	97.20 ± 1.49	0.982 ± 0.75
17	SW	96.02	94.79	95.41	93.97	95.41	0.988
18	SW	95.7	95.17	94.71	95.80	96.89	0.985
19	HS	99.07	98.12	96.23	100.00	100.00	1.000
20	TE	97.8	95.59	93.66	97.69	97.79	0.992
21	WM	99.14	98.27	100.00	97.35	95.24	1.000

^a^Each experiment is for different $\alpha$ and constant $\varphi =0$. Average metrics values are shown.

^b^Each experiment is for different $\alpha$ and random $\varphi$. Average metrics values are shown.

## Results and discussion

Tables 1, 2, 3 specify the performances of the network (accuracy, recall (sensitivity), specificity, precision, and area under the curve (AUC) of the receiver operating characteristic (ROC) graph) for the various experiments based on the testing dataset. All but the TE cells had results higher than 90% in all the metrics, with the TE metrics above 80%. Table 1 includes the experiments done on the synthetic holograms, Table 2 includes the experiments done on the OPD profiles, and Table 3 includes the experiments done on the original raw holograms.

**Table 2:** List of the experiments done on the OPD profiles and the resulting performance metrics.

Experiment number	Cell pair	Final validation accuracy (%)	Test accuracy (%)	Test recall (%)	Test specificity (%)	Test precision (%)	AUC
22^a^	SW	96.48	95.78	95.24	96.53	97.43	0.992
23	HS	92.99	93.90	92.38	95.37	95.10	0.984
24	TE	87.91	84.19	82.07	86.61	87.50	0.940
25	WM	99.57	99.57	100.00	99.32	98.81	1.000
30	SW	96.7	96.39	96.26	96.58	97.43	0.994

^a^Training set included only four of the eight augmentations.

We did not find trends between Experiments 1–8 and between Experiments 9–16 to report, implying the network strength to cope with different fringes spatial frequencies. The main difference between the two sets of experiments is attributed to the randomness of the direction angle, which typically changes less than the off-axis angle.

It arises from Experiments 1–18 (Table 1) that classifying through random angles (size or direction) is more challenging than performing classification based on one angle. In Experiment 18, when training on ImageNet pre-trained network with both random fringe spatial frequency and direction, the process failed, resulting in an accuracy of around 50%. To resolve this, we expanded the dataset to all eight augmentations and used Experiment 17 as the pre-trained network; thus, we achieved good classification results, albeit this test is the most complicated task due to its randomness of the parameters.

**Table 3:** List of the experiments done on the original raw holograms and the the resulting performance metrics.

Experiment number	Cell pair	Final validation accuracy (%)	Test accuracy (%)	Test recall (%)	Test specificity (%)	Test precision (%)	AUC
26^a^	SW	96.84	94.04	93.26	95.16	96.51	0.983
27	SW	95.57	97.35	95.56	100.00	100.00	0.993
28^a^	SW	95.57	94.04	92.31	96.67	97.67	0.969
29^a^^,b^	SW	92.41	91.65	94.42	88.35	90.63	0.976

^a^Transfer learning on Experiment 17.

^b^Training on a test set (10% of the data) only. Metrics are calculated based on the whole train set.

**Figure 3:**
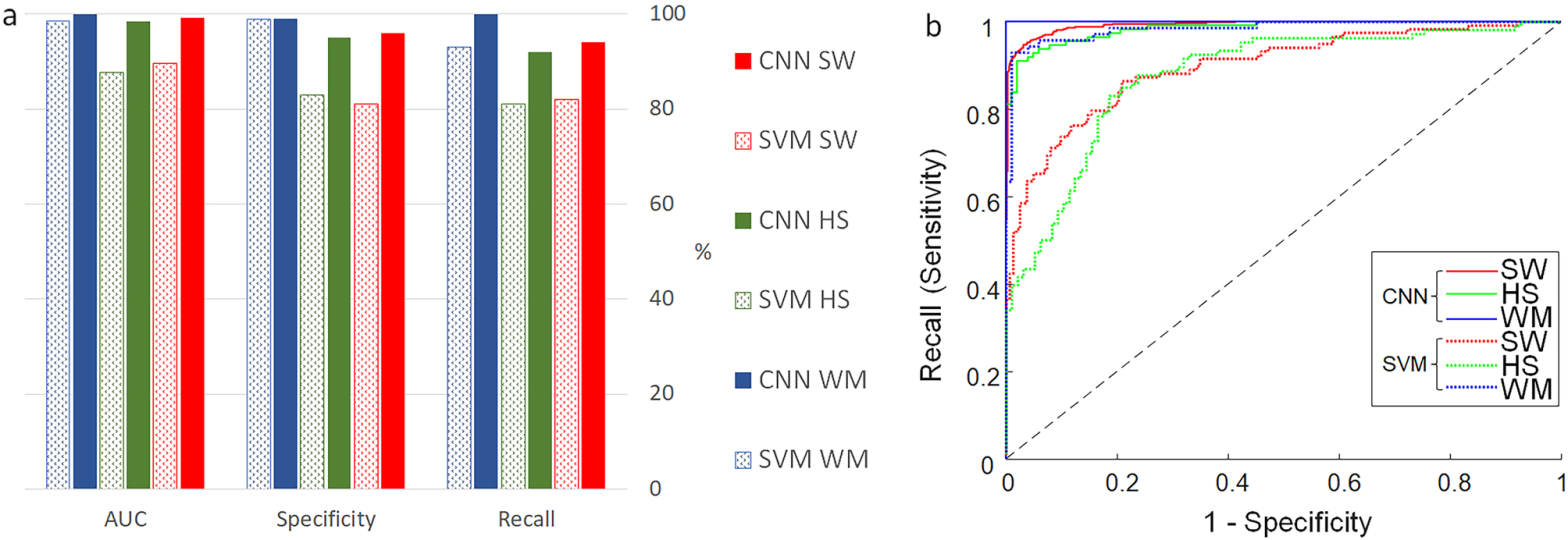
Comparison of classification performance of the SW cell pairs (red), HS cell pairs (green), and WM cell pairs (blue) for CNN classification based on the OPD profiles (solid), and SVM classification based on OPD hand-crafted features (dotted). (**a**) Comparions of AUC, specificity and recall values. (**b**) ROC analysis.

For comparison, we trained the CNN on the calculated OPD and compared the classification performance of the SW, HS, and WM cancer cell pairs (Experiments 22, 23, and 25, respectively) to these obtained in Roitshtain et al.^29^ based on hand-crafted feature extraction followed by applying an SVM. The comparison of the performance metrics along with the ROC analysis are shown in Fig. 3, where the superiority of the CNN to the SVM is clear. In Experiments 18–21, the classification of the synthetic holograms with random angles, both $\alpha$ and $\varphi$, yielded similar or better results to the OPD-based experiments (Experiments 22–25). For better comparison with Experiment 18, we ran Experiment 30 with all eight augmentations on the OPD profiles (Table 2).

Next, we tested the networks trained on the synthetic holograms of the SW cells with the original recorded holograms and got poor results, most probably due to the high contrast of the fringes in the synthetic hologram versus the low fringes visibility in the real low-coherence holograms. Therefore, in Experiments 26–29 (Table 3), we used the captured raw holograms of the SW cells for training with different settings. Experiment 27 is trained, as in previous tests, on pre-trained ImageNet. Experiments 26 and 28 were trained using the network of Experiment 17 with different learning rates. In Experiment 29, we only used the small test set (10% of the dataset) to train the network, based on Experiment 17. The metrics for Experiment 29 were calculated on the whole training set (80% of the dataset), suggesting only a small change to the network weights was enough to utilize the semi-synthetic trained network onto real holograms.

**Figure 4:**
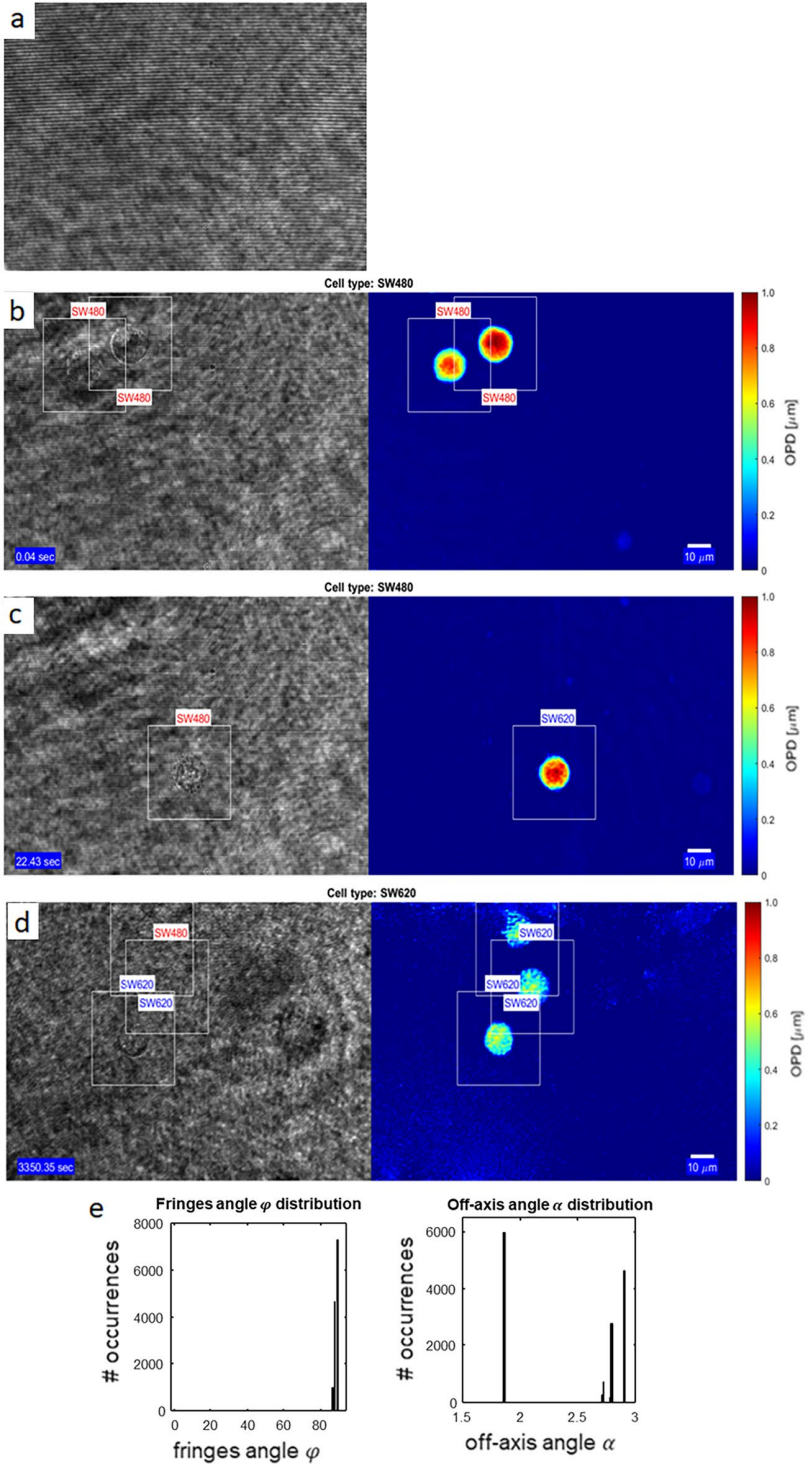
Three example frames from the SW-cell flow videos (see Video 1). Left column presents the original holograms; right column presents the coinciding unwrapped OPD profiles. (**a**) Background hologram without cells. (**b**) Correct detection of SW480 cells; (**c**) Misdetection of an SW620 cell in the OPD-based CNN; (**d**) Correct detection of an SW620 cell, with one misdetection of a cell by the hologram-based CNN; (**e**) Distribution of the off-axis angles and direction angles during the whole video frames, demonstrating the instability of the off-axis angles during the same experiment.

Finally, we used dynamic videos of flowing SW cells in a microfluidic chip and classified them in real-time. We classified them both directly on the raw hologram and on the calculated OPD (Experiment 30) for comparison. The classification was done using the networks trained on the in-focus still images in Experiments 26 and 27. The network in Experiment 26 was trained on the network of Experiment 17, and the network in Experiment 27 was trained on ImageNet weights. The confusion matrices and related metrics are summed in Table 4. Several classification frames are featured in Fig. 4b-d, an example video is presented in Supplementary Video 1. Classification directly on the holograms displayed higher performance than classification on the OPD profiles. The significant difference is most likely due to focusing problems during flow. While the OPD profile quality is very sensitive to focus due to fast changes in the phase around the image plane, the holograms, which are based both on the phase and amplitude, are less affected by changes in focus. The network in Experiment 26 yielded better results, as it was trained on synthetic holograms beforehand (Experiment 17). We chose Experiment 17 as the basis for Experiment 26 since throughout the entire videos (~ 14,700 frames), the fringe direction has not changed much, while the off-axis angle has fluctuated more than a full degree, as shown in Fig. 4e. Calculation of the OPD per frame takes approximatly 20 ms. Classification on the hologram takes 20 ms, while classification on the OPD is done in 25 ms. Thus, even when using deep learning hardware, we could increase the processing time by up to 2.3 times by classifying directly on the raw holograms rather than first processing the holograms to the OPD profiles and only then performing the classification.

**Table 4:** Results for real-time classification of flowing cells.

**Hologram-based classification in Experiment #26**			**Hologram-based classification in Experiment #27**				**OPD-based classification in Experiment #30**		
	Prediction SW480	Prediction SW620		Prediction SW480	Prediction SW620			Prediction SW480	Prediction SW620
SW480	61.96%	5.59	SW480	61.15%	4.62		SW480	27.32%	35.62
SW620	6.66%	25.79	SW620	18.03%	16.20		SW620	1.01%	36.04
Accuracy	87.75%		Accuracy	77.35%			Accuracy	63.36%	
Recall	91.72%		Recall	92.97%			Recall	43.41%	
Specificity	79.49%		Specificity	47.32%			Specificity	97.27%	
Precision	90.30%		Precision	77.23%			Precision	96.43%

## Conclusions

We have developed and trained a CNN to classify live cells during flow directly on the raw off-axis holograms with invariance to the off-axis angle size and direction, affecting the interference fringes of the hologram. First, we demonstrated our approach on semi-synthetic data, synthetic holograms generated based on experimental OPD profiles. We have shown the ability of a common CNN to classify a pair of isogenic cancer cells with different fringe frequencies and fringe directions, yielding similar classification performance to those obtained when classifying the OPD profiles. We have also compared the results of the CNN classification to those obtained by conventional machine-learning classification based on feature extraction. Testing was done by applying transfer learning on experimental holograms, improving their performances.

Furthermore, we have tested the network on dynamic holographic videos of flowing cells. The hologram-based network succeeded in classification and was shown to be less sensitive to cell-focusing problems. Classifying directly on the holograms decreases the calculation time by 2-3 times, speeding up the throughput and robustness of the system. The proposed method provides an important step in the foundation of real-time label-free imaging flow cytometer, enabling actual sorting the further processing for the unlabled cells.

## Supplementary Information

Supplementary Information 1.

Supplementary Information 2.

## Data Availability

All relevant data are available from the corresponding author upon reasonable request.
