# Supplementary material for: On-chip label-free cell classification based directly on off-axis holograms and spatial-frequency-invariant deep learning
Source: Sci Rep. 2023 Jul 31;13:12370. doi: 10.1038/s41598-023-38160-3 (PMC10390541; doi:10.1038/s41598-023-38160-3)
Supplement: Supplementary file 2 — Supplementary Information 2. [file 41598_2023_38160_MOESM2_ESM.docx]

Supplementary video 1 caption:

Example video for classification of cancer cells based directly on the dynamic off-axis hologram, without quantitative phase extraction.
